# Efficacy of low-dose rate brachytherapy utilizing a mold with gold-198 grains for hard palate and gingival cancer

**DOI:** 10.1007/s11282-025-00853-y

**Published:** 2025-08-25

**Authors:** Hitomi Nojima, Atsushi Kaida, Mihoko Haraguchi, Mai Murase, Mariko Hattori, Hirofumi Tomioka, Hiroyuki Harada, Ryoichi Yoshimura, Masahiko Miura

**Affiliations:** 1 Department of Dental Radiology and Radiation Oncology, Graduate School of Medical and Dental Sciences, Institute of Science Tokyo, 1-5-45 Yushima, Bunkyo-Ku, Tokyo, 113-8549 Japan; 2 Department of Advanced Prosthodontics, Graduate School of Medical and Dental Sciences, Institute of Science Tokyo, Tokyo, Japan; 3 Department of Oral and Maxillofacial Surgical Oncology, Graduate School of Medical and Dental Sciences, Institute of Science Tokyo, Tokyo, Japan; 4 Department of Radiation Therapeutics and Oncology, Graduate School of Medical and Dental Sciences, Institute of Science Tokyo, 1-5-45 Yushima, Bunkyo-Ku, Tokyo, 113-8549 Japan

**Keywords:** Oral cancer, Brachytherapy, Gold-198 grain, Mold therapy, Dose simulation

## Abstract

**Objectives:**

Mold therapy using gold-198 grains (Au-mold therapy) is a radical treatment for early stage oral cancer owing to its localized delivery of radiation. Understanding the efficacy and limitations of Au-mold therapy is crucial for optimizing treatment protocols and enhancing outcomes. Thus, this study evaluated the treatment outcomes of Au-mold therapy in patients with hard palate and gingival cancer and assessed its feasibility as a therapeutic modality.

**Methods:**

This retrospective study analyzed data from 37 cases with hard palate and gingival cancer who received Au-mold therapy alone. Treatment outcomes and complications were evaluated, and the dose distribution of Au-mold therapy was simulated using clinically available calculation systems.

**Results:**

A notable 5-year local control rate of approximately 80% was noted in primary cases, whereas recurrent cases exhibited lower local control rates. Severe oral mucositis was not observed, and late complications, such as osteoradionecrosis and oral ulceration, were infrequent following Au-mold therapy.

**Conclusions:**

This study provides insights into the use of Au-mold therapy for oral cancer. Promising outcomes in primary cases suggest its potential as a treatment option. Future research should focus on innovative strategies to enhance the efficacy of Au-mold therapy in oral cancer management.

## Introduction

**Fig. 1:**
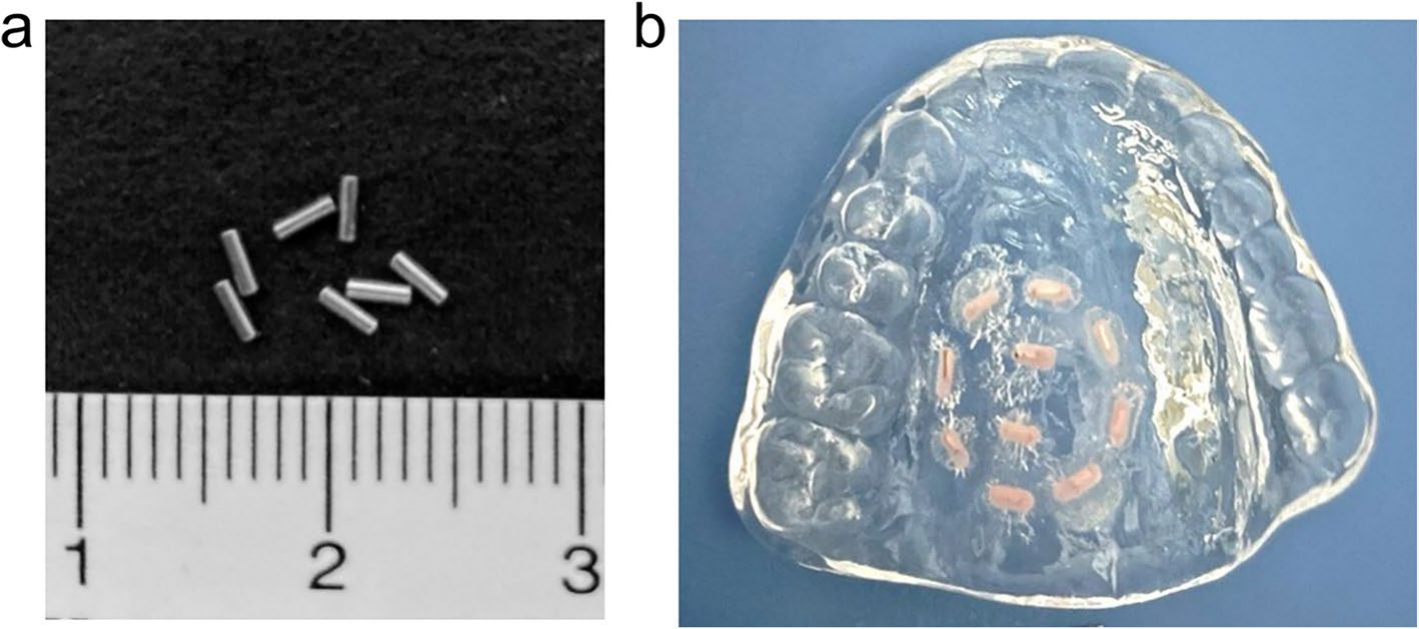
Photos of Au-198 grains (**a**) and a mold (**b**). Ten Au-198 grains (pink) are embedded with paraffin wax and autopolymerizing resin in this mold

Low-dose-rate brachytherapy (LDR-BRT) is an effective treatment for early stage oral cancer [1–3]. In Japan, two primary sources are used for LDR-BRT: grain-shaped gold-198 (Au-198; Fig. 1a), which is suitable for most oral cavity sites, and iridium-192 (Ir-192) pins, which have extensive applications in mobile tongue cancer. Typically, Au-198 grains are directly implanted into tumor tissues under local anesthesia, enabling localized high-dose administration within a week. However, for tumors of the hard palate and gingiva, direct insertion is difficult due to the thickness of the mucosa and presence of bone. Consequently, an alternative approach involves irradiation using a mold resembling a denture, particularly for hard palate and gingival cancers [3]. A customized mold (Fig. 1b) was crafted for each patient, wherein Au-198 grains were embedded (Au-mold), and radiotherapy was administered for approximately 5 d. While isolation is necessary during Au-mold therapy to prevent radiation exposure to others, the procedure is minimally invasive and imposes little physical burden on patients.

Many previous studies demonstrated the effectiveness of mold therapy utilizing Au-198 and Ir-192 sources in early stage mucosal carcinoma of the hard palate and gingiva; however, they primarily included patients who underwent external irradiation in combination with mold therapy [3–5]. Therefore, the efficacy of LDR-BRT using Au-mold therapy alone remains underexplored owing to the limited number of feasible facilities. This study retrospectively investigated the efficacy of Au-mold therapy for hard palate and gingival cancers. Furthermore, in brachytherapy, the irradiated dose decreases sharply with distance from the Au-198 source in accordance with the inverse square law. Therefore, simulating the dose at the peripheral edge of the tumor, where recurrence may occur due to insufficient irradiation, would be important. In this study, dose variations at the tumor periphery were assessed as a function of depth using the current Au-198 grain arrangement in mold therapy, and potential strategies to enhance therapeutic efficacy were explored.

## Materials and methods

### Data collection

Between 2004 and 2022, 86 patients (89 tumor sites) underwent Au-mold therapy at the Institute of Science Tokyo Hospital. Of which, 37 cases with stages I and II squamous cell carcinomas of the hard palate and upper or lower gingiva were selected. All selected cases had received Au-mold therapy alone. The age, sex, site, T classification, treated area, number of Au-198 grains used, dose, and irradiation time were recorded. The T classification criteria were based on the seventh edition of the UICC for International Cancer Control TNM classification for oral cancer [6]. Following Au-mold therapy, all patients were clinically evaluated during follow-up examinations that assessed the local control rate, disease-specific survival rate, rate of cervical metastasis, and time from Au-mold therapy to cervical metastasis. This study was approved by the Ethics Review Committee and Committee on Conflicts of Interest in Clinical Research at the Institute of Science Tokyo (Approval No. D2022-057).

### Au-mold therapy

The mold was fabricated using heat-polymerized acrylic resin [7]. Slots were prepared at the positions wherein the Au-198 grains were arranged (Fig. 1b), which were then covered with wax and an autopolymerizing resin. If necessary, lead sheets were added to the mold to shield the surrounding tissues from radiation. The treated area was calculated according to the tumor size, and the prescribed dose was estimated using the Paterson–Parker system at a position 5 mm away from the Au-198 grains. The actual dose distribution was computed using the Oncentra manual LDR version 1.0 (Elekta, Stockholm, Sweden). To evaluate the dose at the peripheral edge of the tumor base, we assumed that the tumor was cylindrical with a 2 cm-diameter circular bottom, with 10 Au-198 grains evenly distributed within the mold. After which, the dose distribution was calculated using the Oncentra manual LDR, same as the clinically available system.

### Evaluation of complications

To evaluate early and late complications, the severity of oral mucositis was graded according to the Common Terminology Criteria for Adverse Events (CTCAE) version 5.0. In addition, the duration of mucositis was calculated as the number of days from the start of Au-mold therapy to the complete disappearance of the mucositis. To evaluate late complications, the incidence of bone exposure and oral ulcers was observed. In this study, bone exposure was defined as osteoradionecrosis.

### Statistical analysis

Statistical analyses were performed using the GraphPad Prism software (GraphPad Software Inc., MA, USA). Kaplan–Meier plots were compared using the log-rank test. *P* values < 0.05 were considered statistically significant.

## Results

### Patients’ characteristics

We divided the cohort into two groups, primary and recurrent, and characterized them accordingly (Table 1). The cohort consisted of 31 primary cases (T1, 10 cases; T2, 21 cases) and six recurrent T2 cases. The median treated area were 3.8 and 3.3 cm^2^ in primary and recurrent cases, respectively. The number of Au-198 grains, which varied according to the case and median prescribed dose, was approximately 75 Gy in both primary and recurrent cases. The treatment duration ranged from 113 to 216 h (median, 120 h).

**Table 1:** Characteristics of primary and recurrent tumors

		Primary	Recurrent
Total number of patients		31	6
Age (year), median (range)		74 (45–91)	79.5 (54–84)
Gender	Male	13	4
	Female	18	2
Site	Hard palate	7	3
	Upper gingiva	10	2
	Lower gingiva	14	1
T classification	1	10	0
	2	21	6
Treated area (cm^2^), median (range)		3.8 (1.6–7.9)	3.3 (2.1–6.3)
Number of grains, median (range)		11 (7–24)	13.5 (10–25)
Dose (Gy), median (range)		74.7 (67.1–88.6)	74.5 (67.3–86.0)
Irradiation time (hour), median (range)		120 (113–192)	120 (120–216)

### Treatment outcomes of Au-mold therapy for hard palate and gingival cancer

**Fig. 2:**
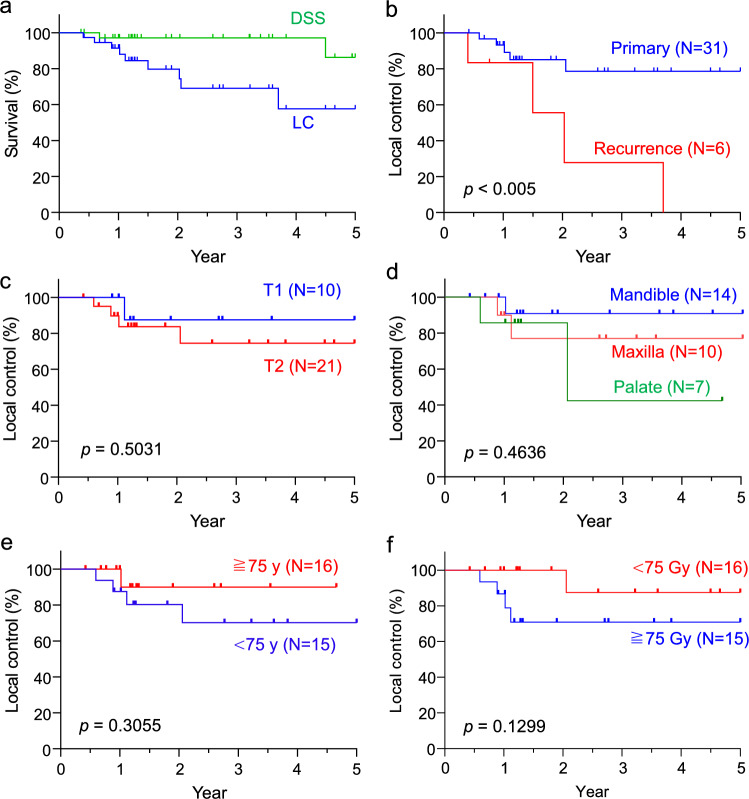
Treatment outcomes following Au-mold therapy. **a** Kaplan–Meier plots of disease-specific survival (DSS) and local control (LC) in all cases. **b** Kaplan–Meier plots of LC in primary and recurrent cases. **c–f** Kaplan–Meier plots of LC grouped according to T classification (**c**), the site (**d**), age (**e**), and treatment dose (**f**) in primary cases. *P* value is calculated by the log-rank test

We evaluated the treatment outcomes in patients undergoing Au-mold therapy. The 5-year local control and disease-specific survival rates were 58% and 86%, respectively (Fig. 2a). Compared with recurrent cases, primary cases reached 79% at the 5-year local control rate (Fig. 2b), indicating higher rates of local control. Data stratification based on T classification, site, age, and treatment dose did not reveal any significant differences in local control (Fig. 2c–f). However, we observed a trend toward higher rates of T1 tumors (T1, 88%; T2, 74%) and cancers located in the lower gingiva (hard palate, 43%; upper gingiva, 77%; and lower gingiva, 91%). In contrast, T2 and hard palate cases exhibited lower local control rates. Additionally, the 5-year cervical metastasis rate was 13.5%, which was lower than that reported in the previous studies on brachytherapy [8, 9]. The median time from Au-mold therapy to cervical metastasis was 443 d, occurring within 2 years, which is consistent with the previous brachytherapy studies [8, 9]. These results suggest that Au-mold therapy, particularly in primary cases, served as a radical treatment approach, although variations in local control rates may arise depending on the tumor site and size.

### Early and late complications after Au-mold therapy

**Fig. 3:**
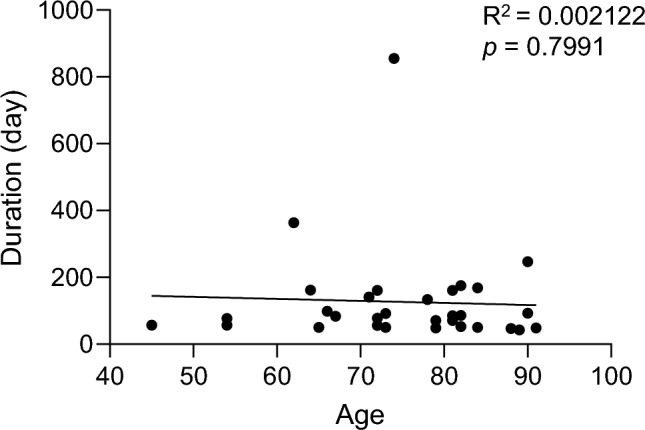
Effect of age on the duration of acute oral mucositis. Correlation between age and oral mucositis duration. Each point represents a value corresponding to an individual patient. No significant correlation was observed (*R*^2^ = 0.002122)

Following Au-mold therapy, oral mucositis commonly occurs at irradiated sites; notably, grade 1 or 2 mucositis was evident in all patients, with no instances of grade 3 mucositis observed (Table 2). The median duration from the initiation of Au-mold therapy to the resolution of oral mucositis was 84 d, which is consistent with a previous study utilizing Ir-192 pins [9]. Considering that an earlier study highlighted a strong association between age and acute toxicity [10], we examined whether the repair time for oral mucositis correlated with patient age. However, no significant correlation was found between duration and age (Fig. 3). Furthermore, late complications, such as oral ulcers and osteoradionecrosis, were occasionally observed following Au-mold therapy. Specifically, oral ulceration and osteoradionecrosis occurred in 11% and 14% of patients, respectively (Table 2). Therefore, while Au-mold therapy commonly induces oral mucositis, a subset of patients may experience late complications.

**Table 2:** Incidences of early and late complications following Au-mold therapy

		Primary	Recurrent
Early complication, oral mucositis	Grade 1	8	0
	Grade 2	23	6
	≧Grade 3	0	0
Late complication, oral ulcer		4	0
Late complication, osteoradionecrosis		5	0

### Evaluation of dose at the peripheral edge of the tumor in Au-mold therapy

**Fig. 4:**
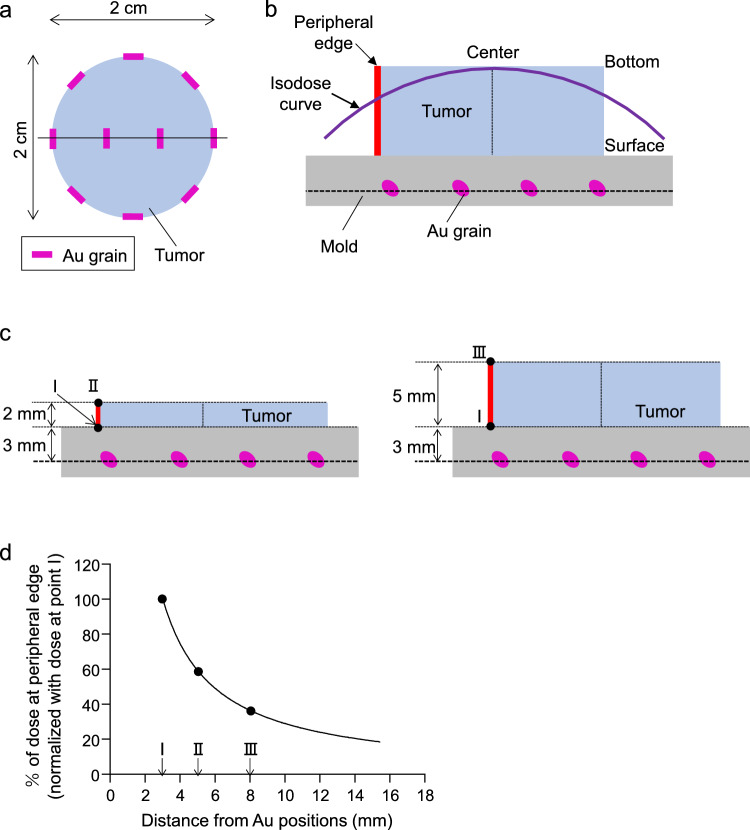
Evaluation of dose on the peripheral edge of the tumor in Au-mold therapy. **a**–**c** Top **a** and sectional **b** and **c** views of the simulated arrangement of Au-198 grains. The tumor shape was assumed to be a cylinder with a circular bottom (2 cm diameter, light blue), and eight and two Au-198 grains (magenta) were arranged in the periphery and center of the area, respectively (**a**; top view). The Au-198 grains were arranged 3 mm from the surface of the mold (**b** and **c**; sectional view). A virtual isodose curve (violet) was shown in b. Tumors with thickness of 2 and 5 mm were illustrated in the left and right panels, respectively (**c**). Points Ⅰ, Ⅱ, and Ⅲ (black) indicate positions 3, 5, and 8 mm from the Au-198 grain on the peripheral edge of the tumor (red line), respectively. **d** Curve represents the relationship between distance and dose percentage at the peripheral edge of the tumor. Each percentage was normalized at 3 mm, where the mold surface was attached to the tumor. An approximate curve was drawn using the normalized values. The percentage on both sides of the peripheral edge was calculated and average value was applied

In our clinical setting with direct implantation, the prescribed dose of 70 Gy was determined at a position 5 mm from the Au-198 grains using the Paterson–Parker system, where they are supposed to be positioned assuming a plane around the tumor bottom in the case of superficial tumors. However, during Au-mold therapy, Au-198 grains are positioned approximately 3 mm from the mucosal surface of the mold [3], on a plane different from the tumor surface. As the dose should be higher at the center of the tumor than at its periphery, it is important to examine how the dose at the edge decreases with increasing distance from the Au-198 grains. In our simulation, we assumed that the tumor would be cylindrical with a circular 2 cm bottom (bottom area: 3.14 cm^2^), with 10 Au-198 grains evenly distributed at a position 3 mm from the tumor surface and embedded in the mold (Fig. 4a–c, dotted line where Au grains are arranged). Figure 4b and c shows cross-sectional views of the tumoral center. All calculated doses were expressed by normalizing the value at a position 3 mm from Au grains to 100%. (Fig. 4c, d-point Ⅰ), corresponding to the interface where the mold contacts the tumor surface. As shown in Fig. 4d, the dose at the peripheral edge decreased exponentially with distance. At distances of 5 and 8 mm from the Au-198 grains (Fig. 4c, d-points Ⅱ and Ⅲ), the dose decreased by 41% and 63%, respectively, suggesting that tumor cells located farther from the mold surface may receive significantly less radiation than those located at the surface. This potentially resulted in underdosing at the tumor periphery when the grains were arranged 3 mm away from the mold surface.

## Discussion

This study represented the first comprehensive analysis of treatment outcomes with Au-mold therapy over 5 years, revealing a local control rate of approximately 80% in early stage primary cases (Fig. 2b). Takeda et al. reported a 2-year local control rate of 74% in patients with oral cancer who had undergone external radiotherapy pre-Au-mold therapy [3]. In contrast, two other studies utilizing high-dose-rate mold therapy with a more potent radioactive Ir-192 source demonstrated 2-year local control rates of 54% and 68%, respectively [4, 5]. Additionally, Au-198 grains showed promising results when directly implanted into tumor tissues such as in tongue and buccal cancers. A previous study reported 2-year local control rates of 86% and 73% in T1 and T2 cases of oral and oropharyngeal cancers, respectively, with direct implantation [1], which is comparable to our results. Notably, Au-mold therapy did not induce severe oral mucositis (Grade ≥ 3), and late complications, such as ulcers and osteoradionecrosis, were infrequent, despite direct irradiation of bones at high doses (Table 2). Thus, Au-mold therapy has emerged as a viable radical treatment option, particularly for primary and early stage hard palate and gingival cancers, with minimal complications. Furthermore, it offers a noninvasive alternative to other forms of brachytherapy, making it particularly suitable for geriatric patients who are unideal candidates for surgery due to their poor performance status and comorbidities. Our experience in applying this therapy to numerous geriatric patients suggests that age does not significantly impact local control or mucositis duration (Figs. 2e, 3), further supporting the use of Au-mold therapy for oral cancer in this population.

Fundamentally, Au-mold therapy is well suited for treating superficial tumors of the hard palate and gingiva that do not involve bone invasion. According to the Paterson–Parker system [3], optimal therapeutic efficacy is expected when tumor tissue lies within approximately 5 mm of the Au-198 grains. Therefore, for superficial tumors up to 2 mm thick, this treatment modality can provide sufficient dose coverage. However, as the distance between the Au-198 grains and tumor cells increase, the delivered dose at the peripheral edge of the tumor base decreased significantly, as demonstrated in our simulation (Fig. 4d). Previous studies have reported mucosal thicknesses ranging from 0.9 to 1.2 and 2.9 to 5.7 mm for the gingiva and hard palate, respectively [11–13]. Thus, in gingival cancers with mucosal invasion, tumor cells are likely to be within approximately 4 mm of the grains, within the effective range. In contrast, in hard palate cancers, vertical tumor expansion may result in tumor thickness approaching or exceeding 5 mm. Compared to that at the surface, the dose decreased by 63% at a depth of 8 mm (Fig. 4c-point Ⅲ). This suggests that, in certain cases, particularly hard palate or larger (T2) tumors, some portions of the tumor may receive an insufficient dose, potentially contributing to the relatively lower local control rates observed in these subgroups.

This study had several limitations. The sample size was relatively small, limiting our ability to assess the impact of factors, such as T classification and tumor site, on treatment outcomes. Additionally, our equipment precludes detailed dose-volume histogram (DVH) analysis, which is crucial for evaluating tumor coverage with a sufficient dose. Future studies should address these limitations by incorporating advanced imaging techniques and DVH analysis to gain deeper insight into treatment efficacy. Not all tumor thickness data were available for this study, limiting our ability to directly evaluate the relationship between tumor depth and treatment efficacy. Nevertheless, our findings underscore the importance of incorporating tumor thickness into treatment planning. Strategies to address this challenge include optimizing grain placement to align more closely with the tumor surface. Alternatively, pretreatment with external beam radiotherapy or chemotherapy to reduce tumor size may enhance the effectiveness of subsequent Au-mold therapy. In conclusion, we demonstrated the potential of Au-mold therapy for oral cancer and confirmed its high local control rate for primary tumors. However, challenges related to tumor thickness and treatment planning must be addressed to further enhance treatment outcomes. Future research should focus on optimizing treatment planning methodologies and exploring innovative approaches to overcome these limitations and improve the efficacy of Au-mold therapy.

## Data Availability

Data will be made available upon reasonable request.

## References

[1] Horiuchi J, Takeda M, Shibuya H, Matsumoto S, Hoshina M, Suzuki S. Usefulness of ^198^Au grain implants in the treatment of oral and oropharyngeal cancer. Radiother Oncol. 1991;21:29–38. 10.1016/0167-8140(91)90338-h. 1852917 10.1016/0167-8140(91)90338-h

[2] Shimizutani K, Koseki Y, Inoue T, Teshima T, Furukawa S, Kubo K, Fuchihata H, Masaki N, Ikeda H, Tanaka Y. Application of ^198^Au grains for carcinoma of oral cavity. Strahlenther Onkol. 1995;171:29–34. 7839302

[3] Takeda M, Shibuya H, Inoue T. The efficacy of gold-198 grain mold therapy for mucosal carcinomas of the oral cavity. Acta Oncol. 1996;35:463–7. 10.3109/02841869609109923. 8695162 10.3109/02841869609109923

[4] Unetsubo T, Matsuzaki H, Takemoto M, Katsui K, Hara M, Katayama N, Waki T, Kanazawa S, Asaumi J. High-dose-rate brachytherapy using molds for lip and oral cavity tumors. Radiat Oncol. 2015;10:81. 10.1186/s13014-015-0390-z. 25888772 10.1186/s13014-015-0390-z PMC4465005

[5] Reshko LB, Gaskins JT, Bumpous JM, Tennant PA, Khan Z, Sowards K, Silverman CL, Dunlap NE. Surface mould brachytherapy in oral and oropharyngeal cancers. Contemp Oncol (Pozn). 2021;25(4):254–63. 10.5114/wo.2021.111087. 35079233 10.5114/wo.2021.111087 PMC8768046

[6] Edge SB, American Joint Committee on Cancer ACS. AJCC cancer staging handbook: from the AJCC cancer staging manual: Springer; 2010. 10.1245/s10434-010-0985-4 20180029

[7] Taniguchi H. Radiotherapy prostheses. J Med Dent Sci. 2000;47:12–26. 12162523

[8] Shibuya H, Hoshina M, Takeda M, Matsumoto S, Suzuki S, Okada N. Brachytherapy for stage I & II oral tongue cancer: an analysis of past cases focusing on control and complications. Int J Radiat Oncol Biol Phys. 1993;26:51–8. 10.1016/0360-3016(93)90172-r. 8482630 10.1016/0360-3016(93)90172-r

[9] Kaida A, Watanabe H, Toda K, Yuasa-Nakagawa K, Yoshimura R, Miura M. Effects of dose rate on early and late complications in low dose rate brachytherapy for mobile tongue carcinoma using ^192^Ir sources. Oral Radiol. 2017;33:187–92. 10.1007/s11282-016-0263-7.

[10] Middelburg JG, Mast ME, de Kroon M, Jobsen JJ, Rozema T, Maas H, Baartman EA, Geijsen D, van der Leest AH, van den Bongard DJ, van Loon J, Budiharto T, Coebergh JW, Aarts MJ, Struikmans H. Timed get up and go test and geriatric 8 scores and the association with (chemo-)radiation therapy noncompliance and acute toxicity in elderly cancer patients. Int J Radiat Oncol Biol Phys. 2017;98(4):843–9. 10.1016/j.ijrobp.2017.01.211. 28366575 10.1016/j.ijrobp.2017.01.211

[11] Nakamura T, Hasegawa A. A study on measurement of gingival thickness in periodontal patients Nihon Shishubyo Gakkai Kaishi. J Jap Soc Periodontol. 2001;43:204–16. 10.2329/perio.43.204.

[12] Nik-Azis NM, Razali M, Goh V, Ahmad Shuhaimi NN, Mohd Nazrin NAS. Assessment of gingival thickness in multi-ethnic subjects with different gingival pigmentation levels. J Clin Periodontol. 2023;50:80–9. 10.1111/jcpe.13723. 36089895 10.1111/jcpe.13723

[13] Ogawa M, Katagiri S, Koyanagi T, Maekawa S, Shiba T, Ohsugi Y, Takeuchi Y, Ikawa T, Takeuchi S, Sekiuchi T, Arai Y, Kazama R, Wakabayashi N, Izumi Y, Iwata T. Accuracy of cone beam computed tomography in evaluation of palatal mucosa thickness. J Clin Periodontol. 2020;47:479–88. 10.1111/jcpe.13254. 31912948 10.1111/jcpe.13254

